# *Cutis laxa* presenting as recurrent ileus

**DOI:** 10.1093/gastro/gou045

**Published:** 2014-07-09

**Authors:** Shishira Bharadwaj, Prakash Shrestha, Tushar D. Gohel, Maninder Singh

**Affiliations:** ^1^Department of Gastroenterology/Hepatology, The Cleveland Clinic Foundation, Cleveland, OH, USA and; ^2^Department of Internal Medicine, Guthrie/Robert Packer Hospital, Sayre, PA, USA

**Keywords:** Cutis Laxa, Gastro-intestinal Symptoms, Ileus

## Abstract

*Cutis laxa* (CL) is a rare connective tissue disorder characterized by phenotypic appearance of loose and redundant skin. CL can be congenital or acquired. Congenital forms include autosomal dominant, autosomal recessive and X-linked recessive. Apart from cutaneous abnormalities, CL can present with visceral involvement. In this article, we report a case of CL presenting as recurrent ileus.

## INTRODUCTION

*Cutis laxa* (CL) or elastolysis or dermatomegaly is a rare connective tissue disorder involving a defect in elastogenesis, characterized by loose, inelastic, sagging skin with reduced recoil [1]. Further, CL can be associated with visceral abnormalities including—but not limited to—emphysema, gastrointestinal diverticula, hernias, genital prolapse, aortic dilatation and tortuosity, bronchiectasis and *cor pulmonale* [2]. CL can be congenital or acquired [3, 4] Congenital forms include autosomal dominant, autosomal recessive and X-linked recessive [5]. Here we discuss a rare presentation of recurrent ileus in a patient with underlying CL.

## CASE PRESENTATION

**Figure 1:**
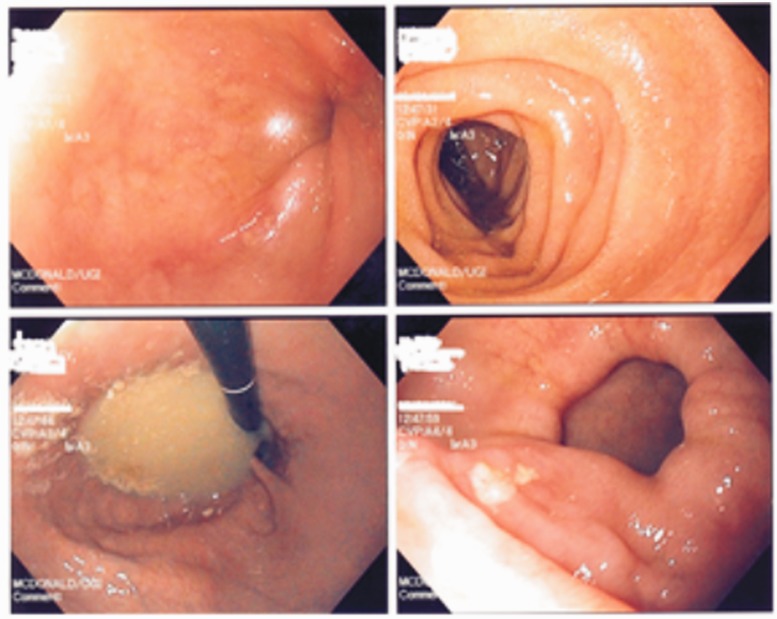
Endoscopic findings of the patients showing lower esophageal diverticulum, patulous stomach, and hiatal hernia.

A 50-year-old female with history of CL diagnosed in 1991, presented to emergency room with complaints of nausea, vomiting and abdominal distension. Her past medical history was significant for bilateral lung transplantation, performed in 2002 secondary to severe emphysema. Review of her system was otherwise unremarkable. Vital signs were stable. Physical examination revealed a distended abdomen with tympanic note on percussion. There were no organomegaly on deep palpation; however, diffuse mild tenderness with absent bowel sounds was noted. Complete blood count and comprehensive metabolic panel were within normal limits. Computed tomography (CT) scan of abdomen and pelvis showed a dilated lower esophagus, focal thickening of the gastroduodenal junction, distended stomach, large hiatal (hiatus) hernia and multiple air-fluid levels in the small bowel suggestive of partial small-bowel obstruction *vs* ileus. The patient was placed on bowel rest; a nasogastric tube was also introduced; fluid and electrolytes were replaced periodically. An upper endoscopy was performed due to the CT scan findings of dilated stomach and thickened gastroduodenal junction. Upper endoscopy showed lower esophageal diverticulum, patulous stomach and a large hiatal hernia (Figure 1). With symptomatic treatment, the patient got better and was discharged; however, the patient continued to experience multiple episodes of ileus, which were managed conservatively. Given the patient's history of CL, phenotypic appearance of loose, sagging skin, upper endoscopic findings of dilated esophagus, patulous stomach and hiatal hernia, the symptoms were attributed to CL.

## DISCUSSION

CL is a rare connective tissue disorder characterized by pendulous, inelastic skin with occasional visceral organ involvement [3]; it could be acquired or congenital [5]. The main histo-pathological finding of CL is deficiency or fragmentation of dermal elastic fibers [6]. Recent advances in research have uncovered the genetic mutations associated with CL [7]; these include—but are not limited to—ATP6V0A2, ATP7A, EFEMP2, ELN, or FBLN5 [8]. The autosomal dominant (AD) type of CL is associated predominantly with skin findings of loose or lax skin due to inelasticity, particularly at neck, armpits and groin and may have normal life span when compared to other subtypes of CL [9]. Autosomal recessive *cutis laxa* (ARCL), which is the most common type of all CLs, includes type I and type II [10]; type II further includes ARCL-IIA and ARCL-IIB [11]. ARCL-I is often associated with severe systemic complications, especially emphysema and diaphragmatic defects, arterial tortuosity and aneurysms, gastrointestinal (GI) and genitourinary complications [12]; ARCL-IIA patients present with more motor nervous system and cardiovascular abnormalities [11]; ARCL-IIB is characterized by skin abnormalities similar to AD [12]; X-linked recessive CL—also called occipital horn syndrome—is characterized by both skin and neurological findings [13]. GI manifestations reported in the literature are more common in autosomal recessive and X-linked recessive and include rectal prolapse, colonic diverticula and pyloric stenosis [14, 15]. Due to the rarity of the disease, the exact incidence of GI manifestations is unknown.

Our case report is the first to describe recurrent ileus, due to decreased motility caused by laxity of the gastrointestinal tract in a patient with CL. Although a biopsy would have been more helpful and definitive, the phenotypic presentation, upper endoscopic findings and CAT scan findings of dilated esophagus, large hiatal hernia, patulous stomach and thickened gastro-duodenal junction suggests that the underlying CL was the likely cause of the patient’s presenting complaints. Further, a percutaneous gastrostomy tube for recurrent ileus could be considered in such patients.

In conclusion, CL is a rare connective tissue disorder with occasional GI manifestations. There is not yet a definitive cure for underlying CL. The prognosis varies with the type of genetic mutation. Genetic counseling can be helpful for patients with family history of CL.

**Conflict of interest:** none declared.
